# Mechanisms of plasticity in a *Caenorhabditis elegans* mechanosensory circuit

**DOI:** 10.3389/fphys.2013.00088

**Published:** 2013-08-23

**Authors:** Tahereh Bozorgmehr, Evan L. Ardiel, Andrea H. McEwan, Catharine H. Rankin

**Affiliations:** ^1^Brain Research Centre, University of British ColumbiaVancouver, BC, Canada; ^2^Department of Psychology, University of British ColumbiaVancouver, BC, Canada

**Keywords:** tap-withdrawal response, *C. elegans*, non-associative learning, habituation, short-term memory, long-term memory, context conditioning

## Abstract

Despite having a small nervous system (302 neurons) and relatively short lifespan (14–21 days), the nematode *Caenorhabditis elegans* has a substantial ability to change its behavior in response to experience. The behavior discussed here is the tap withdrawal response, whereby the worm crawls backwards a brief distance in response to a non-localized mechanosensory stimulus from a tap to the side of the Petri plate within which it lives. The neural circuit that underlies this behavior is primarily made up of five sensory neurons and four pairs of interneurons. In this review we describe two classes of mechanosensory plasticity: adult learning and memory and experience dependent changes during development. As worms develop through young adult and adult stages there is a shift toward deeper habituation of response probability that is likely the result of changes in sensitivity to stimulus intensity. Adult worms show short- intermediate- and long-term habituation as well as context dependent habituation. Short-term habituation requires glutamate signaling and auto-phosphorylation of voltage-dependent potassium channels and is modulated by dopamine signaling in the mechanosensory neurons. Long-term memory (LTM) for habituation is mediated by down-regulation of expression of an AMPA-type glutamate receptor subunit. Intermediate memory involves an increase in release of an inhibitory neuropeptide. Depriving larval worms of mechanosensory stimulation early in development leads to fewer synaptic vesicles in the mechanosensory neurons and lower levels of an AMPA-type glutamate receptor subunit in the interneurons. Overall, the mechanosensory system of *C. elegans* shows a great deal of experience dependent plasticity both during development and as an adult. The simplest form of learning, habituation, is not so simple and is mediated and/or modulated by a number of different processes, some of which we are beginning to understand.

*Caenorhabditis elegans* (*Caeno*, recent; *rhabditis*, rod; *elegans*, nice) is a 1 mm, free-living nematode which was introduced by Sydney Brenner in 1963 as a powerful model organism. During the last 50 years scientists have taken advantage of this tiny creature to reveal functions of genes in developmental and cellular biology. Some of the characteristics which made *C. elegans* an amenable model for doing this research are its small size, short life span, and mode of reproduction. The lineage of the worm's 959 somatic cells was traced through its transparent cuticle, allowing for determination of cell fate (Sulston et al., 1983). Furthermore, *C. elegans* has a sequenced genome comprising approximately 19,000 genes, over 5000 of which have homologues in humans. All of these features contribute to the power of *C. elegans* as a valuable model for understanding molecular mechanisms of cellular plasticity in more complex creatures.

Of the 959 cells in the hermaphrodite worm, 302 are neurons. The wiring and connectivity of the *C. elegans* nervous system has been described (White et al., 1986) and can be divided into a pharyngeal nervous system containing 20 neurons and a somatic nervous system containing 282 neurons. The somatic nervous system contains about 6393 chemical synapses, 890 gap junctions, and 1410 neuromuscular junctions (Varshney et al., 2011). *C. elegans* nervous system is well-adapted to respond to a variety of sensory modalities, including mechanosensation, thermosensation, and chemosensation to mediate behavior (Giles et al., 2006). In 1990, Rankin, Beck, and Chiba were the first to report learning and memory in *C. elegans.* They found that these animals are capable of learning in the form of both short- and long-term habituation. Habituation is defined as a gradual decrease in response to repeated stimuli, which is not explained by sensory adaptation/sensory fatigue or motor fatigue (Thompson and Spencer, 1966). This review focuses on plasticity of the mechanosensory system through the life span of *C. elegans*.

## Mechanosensory circuits

**Figure 1:**
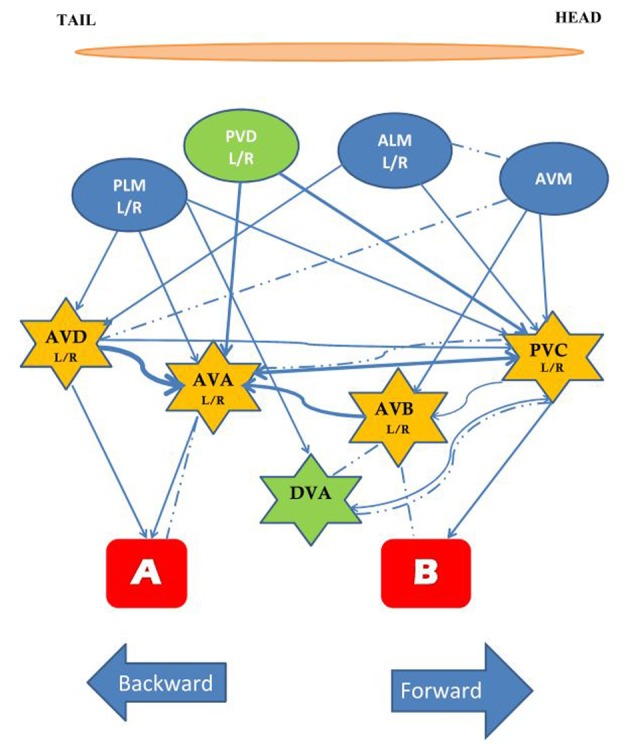
**The circuit mediating the response to tap.** The touch cells are represented by ovals (blue), the interneurons by stars (yellow), and the motor neurons by rectangles (red). All neurons are bilaterally symmetrical, except AVM and DVA. The neurons in green, PVD and DVA, contribute to both forward and backward movement and may play a role in integrating the two responses. Arrows and dashed lines denote chemical and electrical connections, respectively, thickness of lines reflects relative number of synapses [Based on data from Chalfie et al. (1985), Wicks and Rankin (1995)].

*C. elegans* has a variety of sensory neurons that respond to mechanical stimuli. Activity of the touch neurons, proprioceptors, and nociceptors are modulated by mechanical force. Two protein superfamilies' are essential for transforming mechanical stimuli into electrical signals by changing the ionic current in mechanosensory neurons: the TRP channels, and the DEG/ENaC channels. TRP channels are non-specific cation channels composed of six transmembrane alpha helix subunits, while DEG/ENaC channels are predominantly permeable to sodium and in some cases to calcium and consist of two transmembrane alpha helices. The mechanism of mechanotransduction has been broadly studied in *C. elegans* and over 10% of the neurons in the adult hermaphrodite are sensitive to external touch stimuli (Chatzigeorgiou and Schafer, 2011). The best studied of the mechanosensory circuits are the head and tail touch circuits: when a worm is touched lightly on the head it crawls backwards away from the stimulus; when a worm is touched lightly on the tail it crawls forwards away from the stimulus. The touch cell anatomical wiring diagram was determined by serial section electron micrographs (Sulston et al., 1980; White et al., 1986). Subsequently, Chalfie et al. (1985) used laser ablation of neurons to determine the function of cells in the head and tail touch circuits. By killing cells and assaying touch responses Chalfie et al. showed that response to gentle touch to the body was mediated by three mechanosensory neurons in the head (ALML, ALMR, and AVM) and by two mechanosensory neurons in the tail PLML and PLMR. In addition four pairs of interneurons were implicated: AVD, PVC, AVA, and AVB, which are often called command interneurons and integrate information onto motor neurons. Laser ablation showed that AVD and AVA are required for moving backwards to anterior touch, while PVC and AVB are involved in moving forward to posterior touch. The five touch-receptor neurons [ALM (L/R), PLM (L/R), AVM] have a very simple structure. Each cell has a single long process that ends in a synaptic branch. These neurons make synapses at synaptic branches, and along their processes (Chalfie et al., 1985). At the time of hatching ALML and ALMR are located on the left and right side, respectively, in the anterior half of the worm while PLML and PLMR are on the left and right in the posterior. AVM develops post-embryonically and is located in a ventral position (Chalfie and Sulston, 1981). In addition to light touch, the same five mechanosensory neurons respond to a mechanical stimulus delivered to the side of the agar-filled petri plate holding the worm. This vibrational stimulus is called a tap (Rankin et al., 1990) and is thought to simultaneously activate the head and tail touch circuits. In response to tap worms crawl backwards for a short distance. Through laser ablation Wicks and Rankin (1995) confirmed that the five sensory neurons and four pairs of interneurons described in Chalfie et al. (1985) were also critical for the tap withdrawal response. Wicks and Rankin (1995) also hypothesized that DVA and PVD neurons play a role in integrating the sensory input coming from the head and tail (Figure 1). The head and tail input triggers movement in opposite directions; the direction of movement in response to tap is thought to be dependent on imbalanced activity between forward (two sensory neurons) and backward (three neurons) sub-circuits (Chalfie et al., 1985; Wicks and Rankin, 1995). Chiba and Rankin (1990) showed that, before the post-embryonic AVM neuron develops, the circuit is balanced with two sensory neurons for each head and tail, and the behavior consists of 50% forward and 50% backward locomotion in response to tap. When AVM connects to the circuit the bias shifts to reversals (>85% of responses).

## Adult learning and memory: tap habituation

Sensory plasticity plays a critical role in an organisms' ability to regulate processes of attention. Animals are constantly bombarded by sensory information and do not have the attentional resources to attend to all of the inputs at the same time. In response to this, sensory systems have developed the ability to filter out stimuli that are unimportant in that they do not signal appetitive or aversive stimuli. This form of plasticity is the non-associative form of learning called habituation. Habituation is a decrease in responding after repeated presentation of a stimulus, and is distinguished from sensory adaptation and motor fatigue by the ability of a novel or noxious stimulus to rapidly return the response to original levels through dishabituation (only time will allow recovery from adaptation or fatigue). The behavioral characteristics are similar in all organisms that have been studied (Groves and Thompson, 1970). Despite the large number of studies of habituation across a broad range of organisms remarkably little is understood about the mechanisms of this form of learning. *C. elegans* offers a small tractable nervous system and sequenced genome that might facilitate understanding the cellular mechanisms of this “simplest” form of learning. In 1990, Rankin et al. found that repeated taps administered to the side of Petri plates in which worms were cultivated resulted in habituation. They measured reversal distance after each mechanical stimulus and showed that distance decremented after repeating the stimulus 40 times at interstimulus intervals (ISIs) of 10 s or 60 s. This response decrement recovered to the baseline after a few minutes. In these studies, reversal distance and reversal probability were combined into a single measure of response magnitude by assigning worms that did not reverse to a tap a distance score of “0.” To confirm this decrease in reversal magnitude as habituation they needed to rule out sensory adaptation or fatigue by showing dishabituation. To do this they applied electric shock to the agar on which worms were grown. After electric shock a tap resulted in increased reversal distance, indicating that the gradual response decrement to mechanical stimuli could be definitively called habituation (Rankin et al., 1990).

To investigate the locus of short-term habituation Wicks and Rankin (1997) took advantage of the fact that the tap withdrawal circuit significantly overlaps with the thermal avoidance and spontaneous reversal circuits at the level of the command interneurons. They tested whether habituating the tap-withdrawal response had any effect on spontaneous reversals or thermal avoidance responses. Their hypothesis was that if tap habituation training resulted in a decrement of other reversal behaviors, the site of plasticity must be localized to the common circuitry; but if habituation to tap had no effect, the site of plasticity must be at loci unique to the tap-withdrawal response. The result of their experiment showed that tap habituation training had no effect on the frequency or magnitude of spontaneous reversals or on the magnitude of reversals elicited by a thermal stimulus. Based on these results they concluded that the most likely sites of plasticity for tap habituation were the synapses between the mechanosensory neurons and the interneurons or in the mechanosensory neurons themselves.

Adding to the complexity of this story Wicks and Rankin (1996) compared habituation of the head touch circuit with habituation of the tail-touch circuit by laser ablating the tail and head touch cells respectively. They found that the habituation kinetics of the two sub-circuits of the tap response habituated at different rates. Activation of the head touch neurons led to a more gradual decrement of reversals than in intact animals, while activation of the tail touch neurons led first to sensitization then a small amount of decrement of forward accelerations. The kinetics of habituation of intact worms is an integration of these two curves; subtracting the habituation of accelerations from the habituation of reversals produced a curve very similar to that of intact animals. These data suggest that habituation may be mediated by different mechanisms in the head and tail touch neurons. This is an intriguing notion since work on *Aplysia* (Castellucci and Kandel, 1974) led to the prevailing hypothesis that habituation is mediated by modulation of presynaptic neurotransmitter release. Although this hypothesized mechanism has not been identified, it is what researchers expect to find. The idea of multiple mechanisms underlying habituation within a single organism has not been addressed in many studies.

### Short-term memory for tap habituation

To investigate the cellular basis of tap habituation a candidate gene approach was used. To do this, genes expressed in the sensory neurons of the tap circuit were tested for their role in habituation. Gene expression patterns have been determined for a large number of *C. elegans* genes using beta-galactocidase (Fire et al., 1990) or GFP (Chalfie et al., 1994) transgenes expressed by the promoters of candidate genes.

#### Eat-4

**Figure 2:**
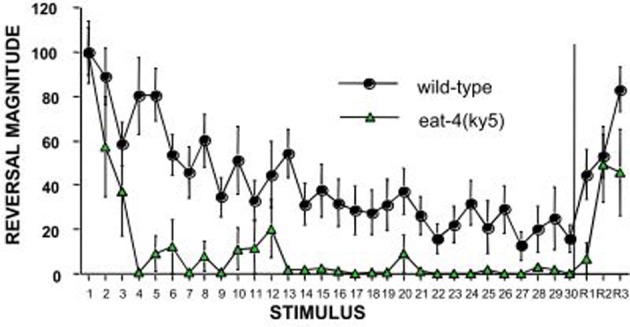
**An example of the candidate gene approach: *eat-4* encodes a glutamate vesicle transporter and is expressed on the touch cells.** Worms with a mutation in *eat-4* show rapid and complete habituation and slower recovery compared to wild-type worms (Rankin and Wicks, 2000).

The *C. elegans* homologue of the mammalian glutamate vesicular transporter (VGLUT1), encoded by *eat-4* expressed in the touch cells ALM, AVM, and PLM (Lee et al., 1999) was the first candidate gene to be tested by Rankin and Wicks (2000). Rankin and Wicks hypothesized that if chemical synapses between the touch cells and the interneurons are glutamatergic then mutations in *eat-4* should cause some deficits in habituation to tap. They found that *eat-4* mutants responded normally to the initial tap, however, they habituated significantly more rapidly and to a deeper asymptotic level than wild-type worms (Figure 2). In addition, *eat-4* mutants did not show dishabituation after receiving a shock following habituation. Reintroducing the *eat-4* gene in the nervous system of the *eat-4* mutant (rescuing *eat-4*) ameliorated the habituation and dishabituation deficits of the mutant. Their findings supported the hypothesis that neurotransmitter release plays a role in habituation and also may play a role in dishabituation (Rankin and Wicks, 2000).

#### Dop-1

Another gene expressed in the mechanosensory neurons (ALM and PLM) is *dop-1*, which encodes a D1-like dopamine receptor (Sanyal et al., 2004). Because studies in a range of species have indicated that the neurotransmitter dopamine plays a critical role in both vertebrate and invertebrate behavioral plasticity, Sanyal et al. (2004) investigated the role of dopamine as a neural modulator in tap habituation. In these studies the authors analyzed reversal probability and reversal distance separately. They observed that *dop-1* mutants showed a more rapid decline in the number of the worms responding to taps during habituation training than the wild-type strain. However, reversal distance during habituation for these mutant animals did not show significant differences compared to wild-type worms. Based on these results, they suggested that dopamine might play a role in modulating habituation to tap. This was the first suggestion that reversal rate and reversal distance habituation might be mediated by different mechanisms (Sanyal et al., 2004).

To determine how dopamine modulates tap habituation in *C. elegans*, Kindt et al. (2007) investigated how the DOP-1 receptors modified activity of the mechanosensory neurons. To address this question, they monitored touch-evoked calcium currents in the mechanosensory neurons. While the initial magnitude of the calcium transients in response to mechanical stimulation was the same in the wild-type and *dop-1* mutants, the transient in the ALM neurons of the *dop-1* mutant animals decreased much faster with repeated stimulation than that of wild-type worms. This effect was rescued by expressing *dop-1* in mechanosensory neurons (Kindt et al., 2007). Interestingly, imaging the posterior touch receptor neurons, PLML and R, revealed that the rate of decrement of touch-induced calcium transients was not changed in *dop-1* mutants compared to the wild-type animals. This suggested that dopamine modulated habituation to tap specifically through anterior touch sensory neurons and also that habituation of these cells was mediated, at least in part, by a gradual decrease in cell excitability. To further elucidate the pathway by which dopamine modulated tap habituation they investigated habituation of candidate signal transduction mutants downstream of DOP-1. DOP-1 is a G protein-coupled receptor, so as a downstream candidate they tested Go (*goa-1*) and Gq (*egl-30*) loss-of-function mutants. The *egl-30* mutant showed a very similar phenotype to *dop-1* for habituation. A mutation in *egl-8* (encodes phospholipase C beta, PLC-β, a putative downstream effector of *egl-30*), also had a habituation phenotype similar to *egl-30* and *dop-1* mutants. Hydrolyzation of PIP2 by PLC-β produces DAG and IP3 and in many systems an important effector of DAG is protein kinase C (PKC). PKC-1 is one of three neuronal PKCs in *C. elegans* and a mutation in the gene that encodes this protein caused rapid habituation similar to that seen in *dop-1, egl-30*, and *egl-8* mutants. Together these results suggested that *dop-1, egl-30, egl-8*, and *pkc-1* encode components of a signaling pathway that modulates the mechanosensory response to tap in ALM neurons (Kindt et al., 2007). One of the more interesting aspects of this study was that this dopamine mediated pathway affected habituation only in the presence of food (*E. coli*), the texture of which was detected by the TRP-4 gentle-touch channel in dopaminergic neurons. In the absence of food there were no differences in habituation between these mutant worms and wild-type worms. This suggests that sensory neuron excitability is modulated by the presence or absence of food—an interesting form of sensory plasticity. The dopamine studies also provide support for multiple mechanisms mediating habituation of a response; the behavioral studies showed response probability was altered by mutations in dopamine genes (while reversal distance was not) and the calcium imaging studies showed that ALM responses were modulated by dopamine, while PLM responses were not.

#### MPS-1

Voltage-dependent potassium channels have been shown to contribute in vertebrate and invertebrate learning and memory (Cohen, 1989; Biron et al., 2006). Phosphorylation of these channels through different signaling pathways can modulate excitability of neurons in response to different external events. However, it was recently discovered that this potassium channel may also have auto-enzymatic activity (Weng et al., 2006). The *kcne* genes in humans encode integral membrane proteins which show kinase activity and can modulate voltage gated K^+^ channels (KVS-1). MPS-1, a member of the KCNE family, is expressed in ALM and PLM neurons of *C. elegans* (Cai et al., 2009). Cai et al. (2009) investigated whether enzymatic activity of MPS-1 acting on K^+^ channels (KHT-1) in mechanosensory neurons play a role in habituation to tap. Applying a single touch to the head or tail of the *mps-1* or *kht-1* mutant animals caused defective backward and forward responses, respectively. The touch response deficit of the *mps-1* mutant could be rescued by expression of wild-type MPS-1 or MPS-1 with an inactive kinase domain. Although they had a wild-type initial response, worms lacking MPS-1 kinase activity habituated more slowly to multiple taps (2, 5, 10, or 60 s ISI; habituation scored as a response magnitude with probability and reversal distance combined), suggesting a critical role of the kinase in the MPS-1 protein for habituation. To explain this result the authors predicted that MPS-1 and KHT-1 form a complex in the touch cells and MPS-1 kinase activity is activated by repetitive stimulation, which results in phosphorylation of KHT-1-MPS-1. Phosphorylation of the KHT-MPS-1 complex decreases K^+^ currents, thereby delaying touch neuron repolarization and decreasing the touch neuron excitability by slowing recovery of voltage gated calcium channels required for signal transduction.

**Figure 3:**
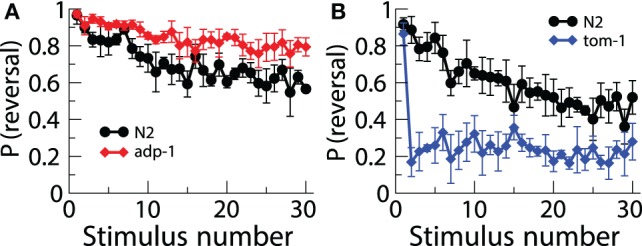
**Using the multi worm tracker to test a number of strains of worms uncovered new mutations affecting habituation.** There was lower response probability habituation to tap in *adp-1* worms **(A)** and greater response probability in *tom-1* worms **(B)** (Swierczek et al., 2011).

Although the first demonstration of habituation in *C. elegans* was published in 1990, and the first gene that played a role in habituation was published in 2000, very few papers have investigated the roles of other genes in habituation. The reason for this slow progress is that most of the experiments tested one worm at a time and then hand scored [or machine scored in Kindt et al. (2007)] one response at a time. Thus, anywhere from 10–40 h were required to run and score habituation for just one strain. Recently a real time computer vision system [Multi-Worm Tracker (MWT)] was designed to allow a high-throughput approach to the study of genes involved in habituation and other behaviors (Swierczek et al., 2011). The MWT is able to analyze image data in real time and monitor many worms on a single plate using a high-resolution camera. Swierczek et al. (2011) used the MWT to conduct a pilot screen for novel genes involved in tap habituation. 33 strains with mutations in genes involved in a variety of predicted functions were habituated and the probability of reversal and reversal distance were analyzed. Among the strains tested was a mutant isolated in a screen for chemosensory adaptation, *adp-1* (Colbert and Bargmann, 1995). Similar to their behavior in the presence of persistent gustatory and olfactory stimuli, *adp-1* mutants maintained robust responding following repeated taps, suggesting a common mechanism between modalities. Chemosensory defective cilia mutants (e.g., *che-2*) also displayed altered tap habituation. The rapid habituation of these mutants may reflect an inability of the dopamine neurons to sense the texture of the bacterial food source and signal to *dop-1* on ALM, but reversal magnitude phenotypes not seen in the absence of food suggests that chemosensory neurons may modulate the tap response. Finally, loss of tomosyn ortholog, *tom-1*, resulted in very rapid and deep habituation (Figure 3). TOM-1 is involved in neurotransmitter release, perhaps linking it to the *eat-4* mutant phenotype. These data demonstrate the usefulness of the MWT as a tool to elucidate mechanisms of tap habituation (Swierczek et al., 2011).

Taken together, these studies suggest that short-term habituation is not as simple as has been thought. These data indicate that habituation is not a unitary process; response magnitude and response probability are mediated or modulated by different genetic pathways. In addition, the head sensory neurons show different patterns of decrement from the tail sensory neurons and are differentially modulated by dopamine. This suggests that different cellular processes may be involved in habituation in different neurons. We also see that short-term habituation is modulated by the presence of food. These studies show that there are increasingly complex aspects to the “simplest form of learning.”

### Long-term memory for tap habituation

Long-term memory (LTM) is defined as the process of saving information in the nervous system that is retrievable over a long period of time. Studies in a number of organisms ranging from human (Ebbinghaus, 1885) to *Aplysia* (Carew et al., 1972) to *Drosophila* (Tully and Quinn, 1985) have indicated that spaced training, in which there are rest periods during training, produces better LTM than massed training (same number of stimuli with no breaks). LTM for habituation of the tap withdrawal response was first shown by Rankin et al. in 1990, and the protocol was replicated and modified in Beck and Rankin (1995) and Rose et al. (2002). The optimal protocol consisted of four blocks of training (1 h rest between each blocks) followed by one block of testing 24 h later. Control groups received one tap at the end of training and one block of test stimuli after 24 h (Rose et al., 2002). Rose et al. showed that as the number of training blocks increased from 1 to 4, the amount of memory also increased. Interestingly, using a 40 min inter-block interval during the training instead of 1 h diminished the significant difference between the control and trained group. Beck and Rankin (1995) showed that applying 45 min of heat shock in the inter-block intervals to block ongoing protein synthesis eliminated memory 24 h later. Heat shock applied in the first or second 15 min in the rest intervals blocked memory, however, if applied for 15 min, 30 min into the block it did not affect memory. Together these data suggest that the first 30–40 min after each block are critical times for memory formation.

A candidate gene approach was used to investigate mechanisms of LTM for tap habituation. Rose et al. (2002) investigated the function of EAT-4, a vesicular glutamate transporter shown to play a role in short-term tap habituation (Rankin and Wicks, 2000) in LTM of tap habituation. Mutations in *eat-4* result in a decrease in glutamate available at the sensory neuron terminal. In *eat-4* worms, spaced training with a tap stimulus did not produce LTM, however, Rose et al. (2002) observed that distributed training with a stronger stimulus, a train of taps produced LTM in *eat-4* mutants. They suggested that stronger stimuli are necessary to release sufficient glutamate from the mechanosensory neurons to produce LTM for habituation in *eat-4* mutants. Based on these data they hypothesized that presynaptic glutamate release from the sensory neurons is necessary for LTM formation (Rose et al., 2002). The necessity of presynaptic glutamate release suggests that postsynaptic glutamate receptors might play an important role in LTM for habituation. GLR-1 is a receptor subunit of a non-NMDA excitatory ionotropic glutamate receptor subtype expressed in the tap circuit interneurons (AVA, AVB, AVDs, and PVC; Hart et al., 1995; Maricq et al., 1995). Although *glr-1* mutants showed normal short-term memory at a 60 s ISI, when tested for LTM of habituation, *glr-1* mutant trained worms were not significantly different from the control group suggesting that *glr-1* was required for LTM formation. To support this hypothesis, a pharmacological experiment in which worms were exposed to DNQX, a non-NMDA-type glutamate receptor antagonist, showed that worms trained in the presence of DNQX did not exhibit LTM. Thus, activation of GLR-1 receptors is critical for memory (Rose et al., 2002).

**Figure 4:**
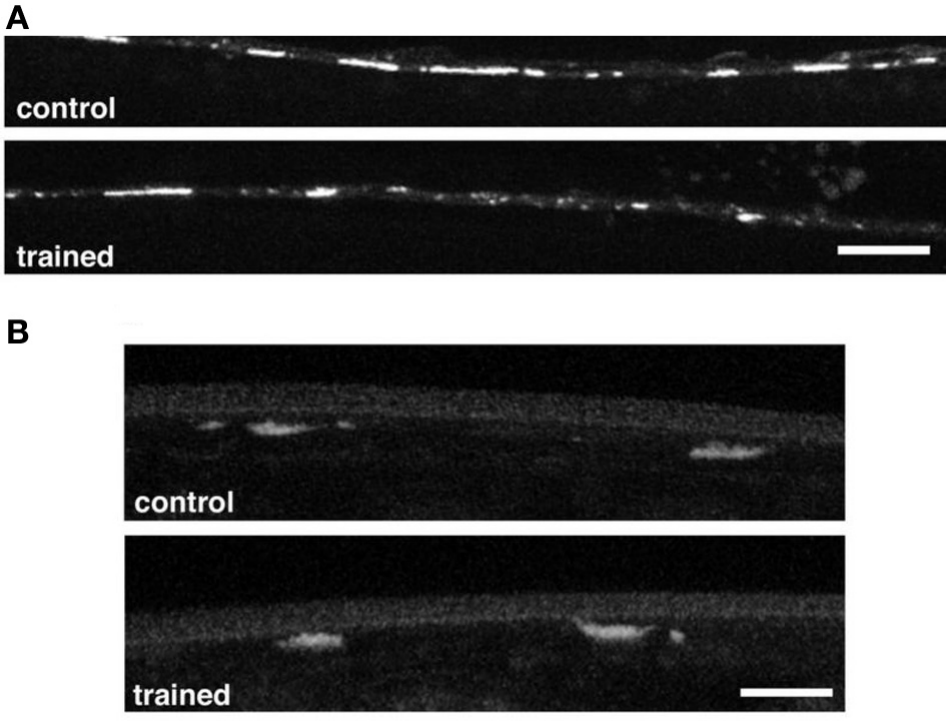
**(A)** Representative images of GLR-1::GFP expression in interneurons of the posterior ventral nerve cord in a worm that had been given spaced training for long-term memory for habituation 24 h before and an untrained control worm. There were significantly smaller GFP clusters in the trained worms than in the control worms. **(B)** Representative images of the vesicles in tap sensory neuron terminals visualized with a synaptobrevin GFP marker in control and trained worms. There was no difference in measured GFP expression between the trained and control worms (Rose et al., 2003).

In *Aplysia* long-term habituation is mediated by a down regulation of vesicles in the siphon sensory neurons (Bailey and Chen, 1991). In mammals LTD (a lasting decrease in synaptic strength similar to long-term habituation) is mediated by down-regulation of AMPA-type glutamate receptors on the post-synaptic neurons. To determine whether LTM training for habituation was associated with changes in the sensory neurons or the command interneurons, worms carrying transgenes with green fluorescent protein fused to pre- and post-synaptic markers were given long-term habituation training and then imaged. One strain expressed GFP marking a synaptic vesicle protein, synaptobrevin (*snb-1*), in the mechanosensory neurons (Nonet, 1999). The other expressed GFP tagged GLR-1 receptor subunits (Rongo and Kaplan, 1999). The results showed no presynaptic changes in GFP tagged *snb-1*, however, spaced training for LTM induced a reduction in the size, but not the number, of GLR-1::GFP puncta in the posterior ventral nerve cord (Figure 4) Rose et al. (2003). Based on these results Rose et al hypothesized that the size of synapses was reduced by memory training, however, the number of synapses was not changed. Interestingly, with more training blocks over several days, decreases were seen in synaptbrevin GFP in the sensory neurons suggesting that plasticity in the sensory neurons requires more stimulation than plasticity of glutamate receptor trafficking in the command interneurons.

### Intermediate memory for tap habituation

In many biological models of learning and memory, researchers have found that, in addition to short-and long-term forms of memory, they also see various forms of memory that they call intermediate memory. These forms of memory do not seem to require protein synthesis and do not last as long as LTM. In *Drosophila* a protein synthesis-independent form of memory (Anesthesia Resistant Memory; ARM) is seen after massed training (Tully and Quinn, 1985). To determine whether massed training could result in an intermediate form of memory in *C. elegans*, Li et al. (2013) looked for the presence of intermediate memory by testing worms 12–16 h after massed training with 80 tap stimuli at a 60 s ISI (No LTM was observed 24 h after massed training, Rose et al., 2002). They found that 12–16 h after massed training, worms did show a significant decrease in reversal magnitude compared to control worms. Thus, intermediate term memory lasts 16 but not 24 h. Investigations into the mechanisms governing this intermediate memory revealed that, unlike LTM, intermediate memory did not require the *glr-1* glutamate receptor subunit. Intermediate-term memory was not affected by applications of heat-shock to block protein synthesis following training (Li et al., 2013).

In order to determine what effect intermediate-memory training had on the tap-withdrawal circuit, worms carrying the GFP tagged *snb-1* expressed in the mechanosensory neurons were given 12 h memory training. Changes in the intensity of the GFP signal would indicate alterations in expression of *snb-1* at the presynaptic terminal. Li et al. (2013) found that, 12 h after habituation training, expression of SNB-1::GFP was significantly higher in trained animals compared to controls. This alteration in the presynaptic terminal was not present 24 h later suggesting that, like the behavioral expression of memory, the modulation at the synapse is transient. These data suggested that 12 h after massed training there was an increase in synaptic vesicles in the sensory neuron terminals. The question then was what is in those vesicles? Normal 12 h memory was found in *glr-1* and *eat-4* mutants indicating that glutamate was not critical for intermediate memory. Using a candidate gene approach, Li et al. (2013) then investigated whether an inhibitory neuropeptide was released by the touch cells. *flp-20* encodes a FMRFamide-related short peptide neurotransmitter precursor with unknown function. *flp-20*, along with *flp-4* and *flp-8*, are the only known peptide precursor genes expressed in the mechanosensory cells (Kim and Li, 2004). Intermediate-memory massed training was given to *flp-4, flp-8*, and *flp-20* mutants and the results showed that only *flp-20* was required for the formation of intermediate memory. Indeed, *flp-20* worms did not show intermediate memory 12–16 h after training, and in a *flp-20* background there was no increase in SNB-1::GFP expression in the presynaptic terminals. Reintroduction of *flp-20* in the mechanosensory neurons rescued the behavioral expression of intermediate memory for massed training. This effect is specific to intermediate memory as *flp-20* mutants were capable of forming LTM after spaced training. Thus, as in *Drosophila*, in *C. elegans* different stimulus paradigms recruit different and independent mechanisms of lasting memory.

### Short- and long-term context conditioning in tap habituation

An organism is not usually exposed to a single stimulus, rather stimuli from multiple sensory modalities occur within a complex environment. Is an animal with a nervous system as small as *C. elegans* able to integrate information across sensory modalities? Historically, simple forms of learning have been divided to two types: associative and non-associative. Habituation, based on this traditional system of classification was placed in the non-associative learning category (Rankin, 2000). However, several studies have suggested that environmental cues have an effect during *C. elegans* training and retrieval of the memory for habituation (Rankin, 2000; Lau et al., 2013). Rankin (2000) showed that worms trained and tested in the presence of a unique context cue (the taste of sodium acetate) showed greater retention of tap habituation 1 h after training compared to a group that did not receive a consistent cue at training and testing. This suggested that worms were capable of associating a chemosensory cue with tap habituation such that after training in the salt taste, the presence of the salt taste cued their habituation memory and test responses were smaller. This is context conditioning for habituation. Lau et al. (2013) replicated the taste context conditioning and extended it to smell, showing that worms trained and tested in the presence of a unique odor cue showed enhanced memory compared to worms that did not receive the cue at both training and testing. Lau et al. used a candidate gene approach to determine whether they could genetically distinguish short-term and LTM for habituation and for context habituation in *C. elegans*. They chose three candidate genes: *crh-1* (homolog of transcription factor, Cyclic-AMP response element binding protein; CREB), *glr*-1 (non-NMDA-type glutamate receptor subunit), and *nmr-1* (NMDA-type glutamate receptor subunit).

Short-term context conditioning experiments were performed by giving 30 taps at a 10 s ISI (habituation training) in the presence or absence of the odorant diacetyl (cue and no cue groups). Because the odorant was on the lid of the Petri plate it could be removed immediately after training. Following a 1-h break, worms were again exposed to the appropriate context (cue or no cue) and given the test stimuli. For LTM experiments, worms were exposed to their randomly assigned context for spaced training (4 or 6 blocks of 20 stimuli at 10 or 60 s ISI with 1 h rest between blocks). Between blocks the plate lids were changed so that worms were not exposed to the odorant during the inter-block interval. 24 h after training worms were tested with 5 taps at a 10 or 60 s ISI either in the presence of the odorant or not. In *C. elegans*, LTM for tap habituation is produced by applying 4–5 blocks of 20 stimuli at 60 s ISI and memory is not detectable if a 10 s ISI is used. Normal LTM for habituation at a 60 s ISI was not enhanced by a context cue, possibly because the 20 min long exposure on each block was enough to produce sensory adaptation, decreasing the salience of the odor cue. Interestingly, LTM for habituation at a 10 s ISI was apparent if worms were trained and tested in the same context. This suggested that the combination of the two sensory modalities produced a new kind of memory not present with the single training cue.

**Figure 5:**
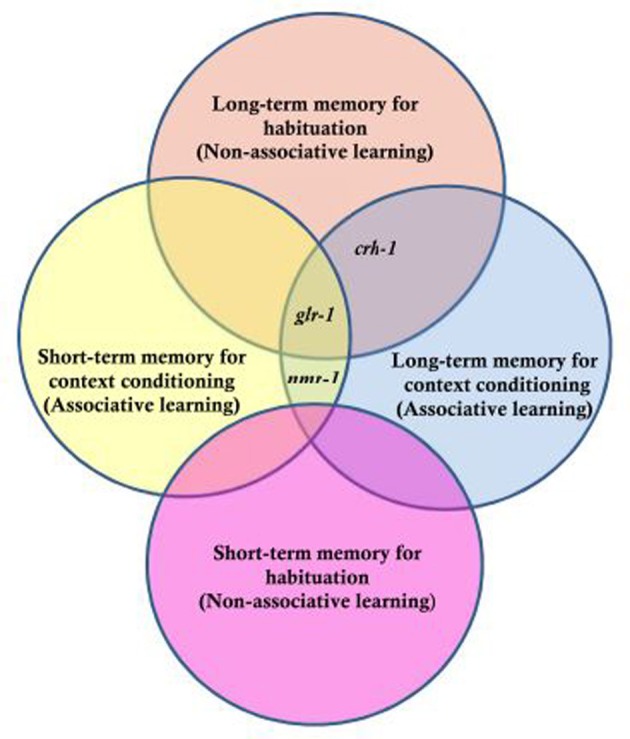
**Genes tested for their role in long- and short-term memory for non-associative habituation and associative context conditioning.** Three genes were tested, *crh-1* which is the worm homologue of CREB, *glr-1* the worm homologue of a glutamate AMPA-type receptor subunit and *nmr-1* the worm homologue of an NMDA-type glutamate receptor subunit. The results indicated that *nmr-1* is required for short and long-term context conditioning but not necessary for non-associative learning; *crh-1* is critical for long-term but not short-term memory for both associative and non-associative memory and that *glr-1* is not required for short-term non-associative learning, but required for long term as well as short- and long-term context conditioning [Based on data from Lau et al. (2013)].

The genetic analyses of short and long-term habituation and context dependent habituation showed that each of the genes tested had a different pattern of results (Figure 5). CREB has been shown to be critical for LTM in various species (e.g., Bernabeu et al., 1997; Josselyn et al., 2004) and worms with a mutation in *crh-1* (homolog of CREB in *C. elegans*) show normal short-term habituation but no LTM (Timbers and Rankin, 2011). For context conditioning experiments, Lau et al. found that worms with a mutation in *crh-1* showed short-term habituation and short-term context conditioning but no long-term context conditioning. Rescuing CRH-1 in mutant worms rescued the long-term context conditioning deficit. In previous studies, it was shown that *glr-1* (encoding a non-NMDA-type glutamate receptor subunit) is critical for LTM performance, but not necessary for short-term memory (Rose et al., 2002). Lau et al. found that in addition to not showing long-term habituation, *glr-1* mutants did not show short-term or long-term context conditioning. *nmr-1* encodes an NMDA-type glutamate receptor subunit broadly expressed in interneurons of the mechanosensory circuit in *C. elegans*. Lau et al. (2013) showed that an *nmr-1* mutant did not show any deficit in short-term or LTM for habituation, however, NMR-1 was critical for both short- and long-term context effects in habituation. How is the input from the two sensory systems integrated? Previous studies showed that expression of *nmr-1* in the pair of RIM interneurons was essential for starvation and taste association learning (Kano et al., 2008). Based on this, Lau et al. (2013) investigated the role of *nmr-1* in RIM interneurons by cell-specific rescue of this gene and found that expression of NMR-1 in the RIM interneurons was sufficient to rescue both short- and long-term context conditioning in *nmr-1* mutant animals. This suggested that the interneuron RIM is a key site of integration for sensory input from chemosensory and mechanosensory neurons. Context conditioning learning in the mechanosensory circuit of *C. elegans* offers a new opportunity to investigate the genes critical for short- and long-term associative and non-associative learning as well as to study how sensory integration is encoded in the memories.

These experiments offer another example about how complex habituation can be, even in an animal with only 302 neurons. *C. elegans* behavior can be affected by previous experience and the response to tap can be altered by the memory of previous training. *C. elegans* is also capable of processing information from multiple sensory systems simultaneously and of forming an association of those stimuli that influences later memory. For short-term habituation, a chemosensory context cue enhances the memory of habituation training 1 h before. For LTM for context conditioning, worms show memory after a tap protocol that would not normally lead to memory. This suggests that the association of tap habituation in the presence of a chemosensory cue led to production of a novel type of memory. In mammals, multiple cues often lead to better memory formation than a single cue (Walker et al., 2005; Marschner et al., 2008). These findings suggest that in every non-associative learning paradigm there is the potential for associative learning to be occurring, as well as for the effects of previous training to influence the behavior. Once again the simplest form of learning gets additional layers of complexity.

## Developmental experience dependent sensory plasticity

So far, this review has focused on the behavioral characteristic and molecular mechanism of short-term, intermediate-term and LTM in adult *C. elegans*. However, mechanosensory stimulation during development can also have a profound effect on adult sensory systems and behavior (Rose et al., 2005). As in mammals (Diamond et al., 1966), activity-dependent processes shape the final patterns and strengths of synaptic connections in the worm's nervous systems (Peckol et al., 1999; Zhao and Nonet, 2000; Rose et al., 2005).

In *C. elegans*, neural wiring and connectivity has been described and was originally suggested to be invariant between individuals (White et al., 1986), nevertheless further research has revealed that activity in the nervous system might be necessary for normal axonal branching and neuronal morphology (Peckol et al., 1999; Zhao and Nonet, 2000). Rose et al. (2005) examined the influence of mechanosensory stimulation that comes from contacts with other worms during development. In the laboratory worms are usually grown in groups in small Petri plates, however, in this experiment, worms were raised either in groups (colony condition) or one worm to a plate (isolated condition). When 4 day-old worms were examined, those that had been raised in the isolated condition showed significantly smaller responses to a single tap compared to the worms that developed in a group. To test the possibility that isolation affected sensory systems in general, Rose et al. looked at different behaviors mediated by the same interneurons and motor neurons as the tap withdrawal circuit. The magnitude of the reversal response to a heat probe was indistinguishable between isolate and colony-reared worms. This suggested that the decrease in reversal behavior to tap in isolated worms resulted from the lack of mechanical stimulation during larval development. Consistent with this hypothesis, thirty tap stimuli administered at any larval stage reversed the effect of isolation on the tap response in adult worms (Rose et al., 2005; Rai and Rankin, 2007).

Rose et al. (2003) found that the GLR-1 glutamate receptor subunit was critical for LTM for habituation training in adult worms. Based on this finding, Rose et al. (2005) hypothesized that GLR-1 function might be important for the effects of isolation on the tap-withdrawal response. To test whether *glr-1* played a role in the decreased response to tap in isolated worms, the magnitudes of reversal responses to tap for the colony and isolate groups were measured in *glr-1* mutants and in a transgenic strain in which *glr-1* expression had been rescued with the endogenous promoter. There were significant differences in response magnitudes between isolate- and colony-raised worms in the wild-type strain and the *glr-1* rescue strain, however, isolated- and colony-raised *glr-1* mutants did not show any difference in the reversal response to tap. Based on these results, it appeared as though the decreased response to tap seen in isolated worms was mediated by the *glr-1* type glutamate receptor subunit.

Rose et al. (2005) used GFP to show that changes in the expression of *glr-1* accompanied the formation of LTM for habituation in adult *C. elegans*, while changes were not seen in the expression of *snb-1* in the mechanosensory neurons. The same GFP constructs were used by Rose et al. (2005) to determine whether there were anatomical changes underlying the effect of isolation on the response to tap. To test whether the amount of stimulation during development altered the expression of *glr-1* receptors, Rose et al. again imaged the GLR::GFP strain (Rongo and Kaplan, 1999). Similar to what was seen in adult memory they found that the number of the GLR-1::GFP clusters in isolate-reared worms was not different from colony-reared worms, however, the size of the clusters was significantly reduced. As with the behavior, brief stimulation (30 taps) during any larval stage was sufficient to increase the level of *glr-1* expression in the interneurons of isolated worms (Rose et al., 2005; Rai and Rankin, 2007). Rose et al. also examined transgenic colony and isolate worms expressing *snb-1*-GFP (SNB-1::GFP; Nonet, 1999), in the touch cells. The expression of SNB-1 in isolated-raised worms was lower than expression in colony-raised worms, which suggested that the number of synaptic vesicles in the mechanosensory neuron terminals was reduced. Thus, mechanosensory deprivation during development appeared to lead to the production of weaker synapses with fewer vesicles in the sensory neurons and fewer receptors on the interneurons than in colony reared animals. Synabtobrevin expression in isolated worms could only be rescued by brief mechanical stimulation applied at the first larval stage (L1) (Rai and Rankin, 2007). Rai and Rankin (2007) showed that as worms aged, progressively more mechanosensory stimuli were required to rescue *snb-1* expression in isolate reared worms to colony-reared levels (i.e., 400 stimuli in L2 or L3 and 800 stimuli in L4 and adult). Again, these results suggest that trafficking of glutamate receptors responds more rapidly to experience than does a presynaptic vesicle protein. The results of these experiments also revealed a strong association between rescuing the response to tap and postsynaptic *glr-1* expression in isolated animals. This suggested that tap reversal behavior is tightly related to glutamate receptor expression. In other words, changes in glutamate receptor expression may underlie the observed changes to the behavioral response to tap (Rai and Rankin, 2007).

The study on the role of early experience on the tap response also led to insight into the mechanisms governing the rate of habituation. When isolate-reared worms were given 30 mechanosensory stimuli at any stage of development the initial response to tap and the level of *glr-1* were similar to colony-reared worms, however, this “rescue” was deceptive, as the “rescued” worms habituated differently than the colony-reared worms. Three groups of adult worms were given habituation training: an isolated group, a colony group, and a rescued isolated group that had been given sufficient stimulation during larval development to rescue the GLR-1::GFP, but not the *snb-1* GFP (Rose et al., 2005). The results showed that the isolate group had significantly smaller initial responses to tap than either the colony or the rescued isolate group, while the rescued isolate and the colony groups were not different from one another. However, with repeated taps, the rescued isolate group showed much more rapid decrement than did the colony group. In fact, the rescued isolates habituation curves looked very like the habituation curves for the *eat-4* worms (Figure 2). The isolated worms had lower *snb-1* expression in the terminals of their touch cells and similarly, *eat-4* worms had a defect in the loading of glutamate in vesicles, suggesting they also had less glutamate available for release. These data suggest the hypothesis that regulation of neurotransmitter release plays an important role in regulating rate of response decrement during habituation. This hypothesis is supported by the finding that another gene involved in regulating neurotransmitter release, *tom-1*, also showed a similarly altered rate of habituation to tap (Swierczek et al., 2011).

### Changes in tap habituation with aging

**Figure 6:**
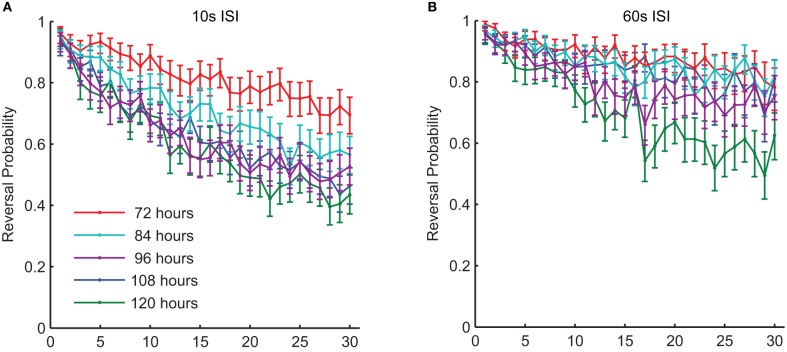
**There were changes in habituation of response probability throughout middle-age in wild-type worms.** This graph shows response probability habituation for 72-, 84-, 96-, 108-, and 120-h-old (all reproductive adults) wild-type *C. elegans* in response to a series of 30 taps. Age was significantly related to the probability of responding to the final tap at both a 10-s ISI **(A)** and at a 60-s ISI **(B)** (Timbers et al., 2013).

In addition to experience dependent changes during development, the ability to learn and remember also alters with normal aging. Beck and Rankin (1993) studied aging *C. elegans* (10 and 12 days post-hatching) and found that they habituated more rapidly and showed less spontaneous recovery than middle-aged adults. This is not unexpected as *C. elegans* rapidly begins to show nervous system and muscle breakdown after it has passed its reproductive prime (Pan et al., 2011). However, what about during middle age, before the neurons and muscles show evidence of degeneration? Are there changes in sensory plasticity during this period? Worms develop from fertilized egg to reproductive young adult in 3.5 days; they then are reproductive adults for 3–4 days, before becoming senescent (Wood et al., 1980). Timbers et al. (2013) investigated whether there were age-dependent changes in habituation in middle-aged *C. elegans*. They tested the effect of age on tap habituation in populations of worms 72 (day 0 of adulthood), 84, 96, 108, and 120 (day 2.5 of adulthood) h old. They found that habituation of reversal probability to tap in young animals (72 h old) occurred more slowly than for older adult (120 h) animals (Figure 6). The factors responsible for this difference between 72 and 120 h old worms might be in the transduction of the tap stimulus or might reflect some change in cell excitability or synaptic strength as worms aged. To investigate this Timbers et al. used transgenic worms expressing Channelrhodopsin-2 (blue light-gated cation channel) in the touch cells (Nagel et al., 2003; Boyden et al., 2005). Applying a short pulse of blue light to the transgenic worms activates the touch cells and induces a reversal response similar to that seen following a mechanical tap. If activation of the touch cells by blue light showed the same results as applying taps in adult worms (72, 84, 96, and 120 h old), the age-dependent changes in behavior must originate downstream of sensory transduction. However, the results of this experiment showed that age was not related to the probability of responding to repeated blue light pulses, suggesting that the age-dependent changes in behavior originate upstream of cellular depolarization. A known characteristic of habituation is that less intense stimuli cause more habituation, whereas more intense stimuli cause less habituation (Thompson and Spencer, 1966; Groves and Thompson, 1970; Rankin et al., 2009). Consistent with this body of literature, Timbers et al. (2013) also showed an association between stimulus intensity and habituation of young adult worms (72 h-old), however, older worms showed no such association. Thus, the rate of reversal probability habituation is dependent on the ability to discriminate stimulus intensity, which the younger worms seem to do to a greater extent than the older worms. Possible explanations for the decrement in stimulus discrimination with age include changes in neuronal excitability, changes in the thickness of the cuticle or in the strength of the connection of the mechanosensory cells to the body wall (Timbers et al., 2013).

## Conclusions

Despite its apparent simplicity, the *C. elegans* tap withdrawal response is a complicated behavior that shows a great deal of plasticity. External mechanical stimuli activate forward and backward sub-circuits and imbalanced activity of the forward and backward sub-circuits determine the direction of the movement. This response is experience dependent and plasticity of the circuit can alter behavior for days. Thus, far tap habituation has shown short- intermediate and LTM—a surprising catalog of types of memory for an organism that only lives a few weeks. A number of factors can influence habituation and the memory for habituation, these include the presence or absence of food, the presence of absence of contextual cues, the amount of early mechanosensory stimulation that particular worm had during larval development, and the age of the worm at the time of testing. In addition different neurons in the circuit are affected by different factors and habituate at different rates. The search for the mechanisms underlying habituation will have to take all of these variables into account, rendering this so-called “simple” form of learning not so simple after all.

### Conflict of interest statement

The authors declare that the research was conducted in the absence of any commercial or financial relationships that could be construed as a potential conflict of interest.
